# Palliative Radiotherapy and Corticosteroid Management for Tracheal Stenosis and Superior Vena Cava Syndrome Caused by a Large Mediastinal Mass Due to Adult T-cell Leukemia/Lymphoma: A Case Report

**DOI:** 10.7759/cureus.83537

**Published:** 2025-05-05

**Authors:** Satoshi Anai, Takeaki Kusada, Kohei Isa, Rin Chibana, Yoko Sato

**Affiliations:** 1 Division of Respiratory Medicine, Yuuai Medical Center, Tomishiro City, JPN; 2 Radiation Oncology, Yuuai Medical Center, Tomishiro City, JPN

**Keywords:** corticosteroids, large mediastinal mass, malignant lymphoma, palliative radiotherapy, superior vena cava (svc) syndrome, tracheal stenosis

## Abstract

Superior vena cava (SVC) syndrome is a clinical condition caused by the compression or obstruction of the SVC, often due to malignant tumors in the mediastinum; it presents with facial edema, upper limb swelling, and dyspnea. We report the case of an 85-year-old man presenting with rapidly progressing SVC syndrome and airway stenosis due to a large mediastinal mass, later identified as malignant lymphoma. Urgent hematology evaluation proved to be difficult, and airway compromise posed immediate clinical challenges. Cytology and positive HTLV-1 serology suggested adult T-cell leukemia/lymphoma (ATLL). Due to rapid progression, palliative radiotherapy combined with corticosteroids was initiated promptly, resulting in a significant reduction of the mediastinal mass and rapid symptom improvement. This report highlights an effective interim strategy for stabilizing patients with massive mediastinal lymphoma presenting with SVC syndrome when immediate hematology referral is unavailable.

## Introduction

Patients with large mediastinal tumors secondary to malignant neoplasm are at high risk of superior vena cava (SVC) syndrome. It occurs when tumor infiltration into the vessel wall or internal obstruction by a tumor thrombus increases venous pressure in the upper body, resulting in congestion of the head and upper limbs. As the condition progresses, it can cause narrowing of the airway and lead to airway obstruction [1]. Approximately 75% of SVC syndrome cases are caused by lung cancer. Among lung cancer subtypes, non-small cell lung cancer accounts for 50%, and small cell lung cancer accounts for 25%. Non-Hodgkin lymphoma and metastatic lung lesions each account for approximately 10%. Hodgkin lymphoma, esophageal cancer, thyroid cancer, germ cell tumors, leukemia, and others account for the remaining cases [2,3].

We previously reported a case of mantle cell lymphoma complicated by pulmonary tuberculosis and another case of T-cell malignant lymphoma mimicking interstitial pneumonia [4,5]. As these reports illustrate, while malignant lymphoma originating in the lungs or mediastinum is extremely rare, it can present with a wide range of symptoms that complicate both diagnosis and treatment. For malignant lymphoma, achieving an accurate histological diagnosis before initiating therapy is essential. Prompt initiation of immunochemotherapy following diagnosis is generally recommended to improve outcomes. However, in cases involving very elderly patients or when immediate referral to a hematology department is difficult, disease management becomes significantly more challenging. We present the case of a very elderly patient with a massive mediastinal lesion leading to SVC syndrome and critical airway stenosis.

## Case presentation

**Figure 1:**
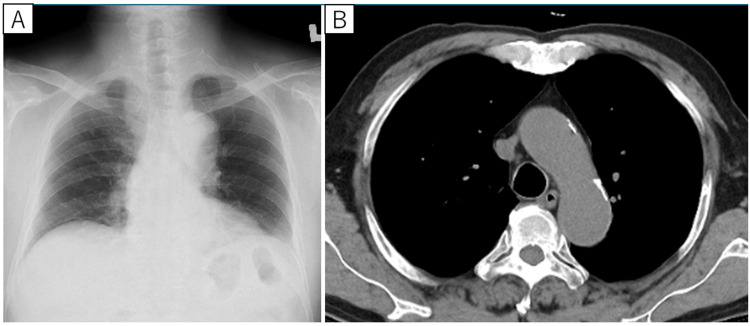
Postoperative chest imaging performed by the Department of Surgery following appendiceal cancer resection, with no respiratory abnormalities noted at that time A postoperative chest X-ray obtained six years prior to symptom onset (A) showed no mediastinal enlargement. Similarly, a chest CT scan performed one year and eight months before symptom onset (B) revealed no apparent abnormalities, including lymph node enlargement CT: computed tomography

The patient was an 85-year-old male who had undergone an open lower cecal resection and D2 lymphadenectomy for appendiceal cancer at the age of 75 at our hospital’s Department of Surgery. The postoperative diagnosis was well-differentiated adenocarcinoma [T1 N0 M0, stage I according to the Union for International Cancer Control (UICC) seventh edition TNM staging system]. He was followed up in the surgical outpatient clinic, and no clear recurrence was noted (Figures 1A, 1B).

**Figure 2:**
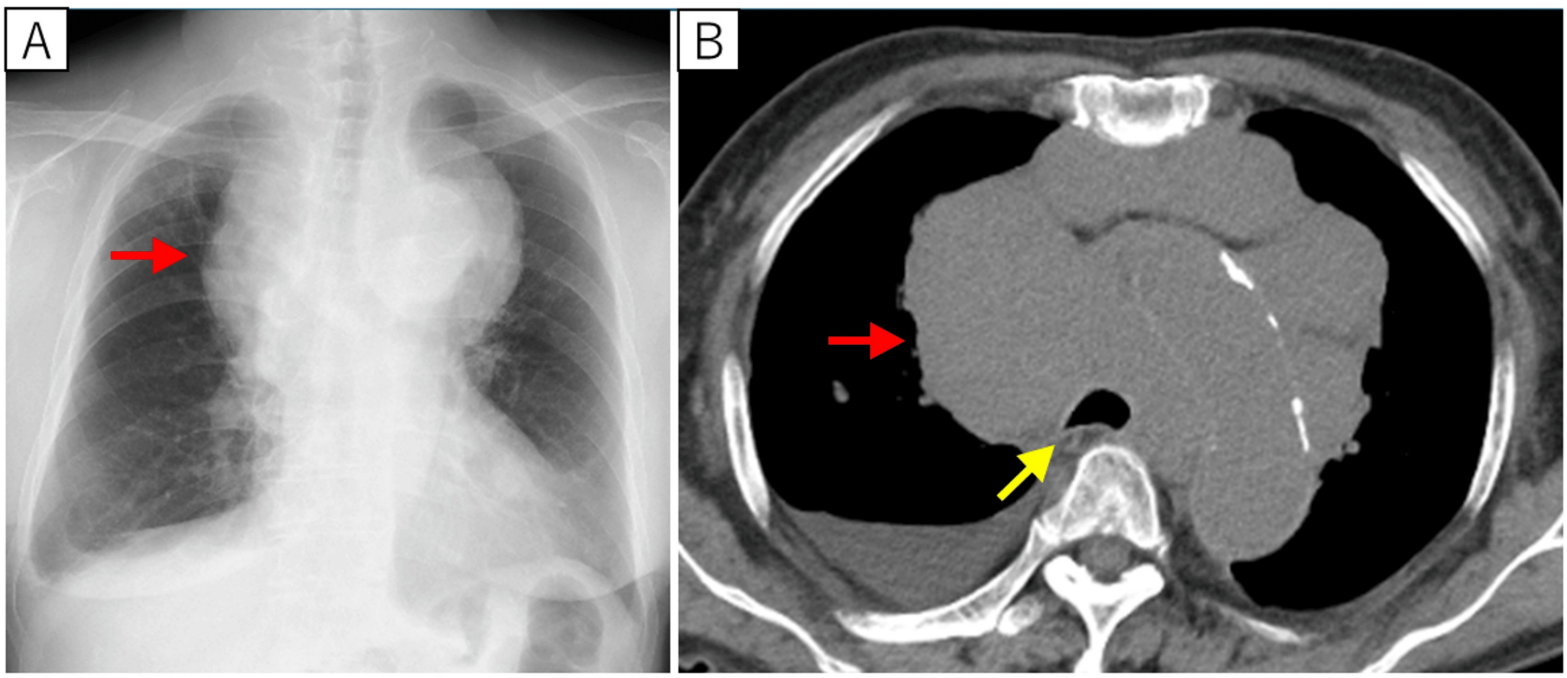
Chest imaging obtained at the onset of superior vena cava syndrome and respiratory symptoms On the chest X-ray (A), a new mass approximately 14.5 cm in length was found in the mediastinum (red arrow). On the chest CT, a mass was found in the mediastinum (red arrow), and the trachea was compressed and narrowed (yellow arrow) CT: computed tomography

Three weeks before referral to our department, a chest X-ray and CT scan had revealed newly enlarged hilar, mediastinal, and cervical lymph nodes, as well as compression of the trachea by the same lesion (Figures 2A, 2B).

Simultaneously, the patient developed a sudden worsening of facial and bilateral upper-limb edema, along with increasing dyspnea and dysphagia. A new right-sided pleural effusion was also observed. Given these imaging findings, malignant lymphoma was strongly suspected. Although a referral to a hematologist was planned, the patient’s anterior neck swelling rapidly worsened, and his dyspnea and dysphagia became more severe. These findings were consistent with SVC syndrome and tracheal stenosis secondary to a large mediastinal mass. Because of the risk of asphyxiation, we considered tracheal stenting or urgent hematology consultation. However, no facility was immediately available for either intervention, and hence the patient was admitted to our hospital’s Department of Respiratory Medicine.

**Figure 3:**
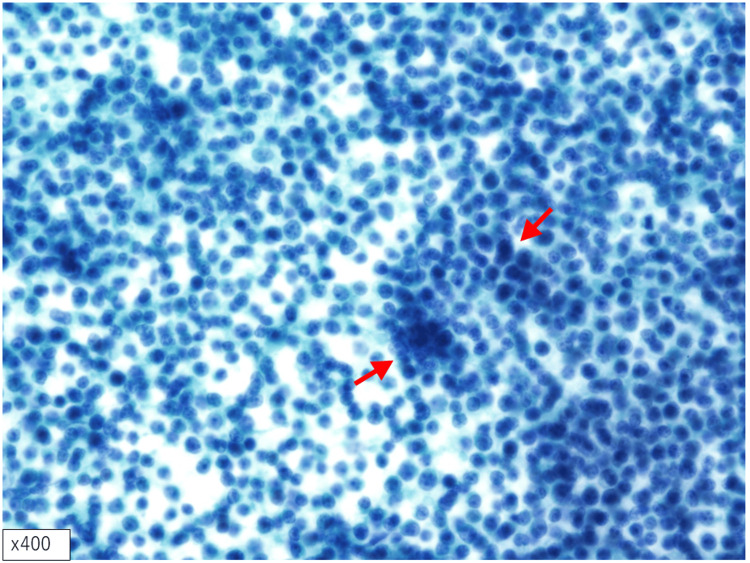
Cytological examination A high nuclear-to-cytoplasmic ratio was observed, with some areas showing atypical cells resembling naked nuclei. These atypical cells displayed solitary proliferation, nuclear enlargement, irregular nuclear contours, and prominent nucleoli (red arrow). Although clustering of the atypical cells was present, no evident intercellular adhesion was observed, supporting a diagnosis of malignant lymphoma (Papanicolaou staining, original magnification, × 400)

On admission, physical examination revealed enlarged, soft, and elastic cervical and supraclavicular lymph nodes without significant tenderness. Although obvious stridor was not detected on auscultation, the patient was unable to lie flat due to worsening dyspnea, raising concern for potential acute airway compromise. Fine-needle aspiration cytology of the right supraclavicular lymph node identified atypical cells arranged singly and lacking epithelial junctions, suggesting malignant lymphoma (Figure 3).

The patient also tested positive for HTLV-1 antibodies, had hypercalcemia, high lactate dehydrogenase (LDH) level (342 IU/L), and had a markedly elevated serum IL-2 receptor (IL-2R) level of 25,000 U/mL. Peripheral blood flow cytometry revealed no specific abnormal cell populations. However, based on the positive HTLV-1 serology and hypercalcemia, adult T-cell leukemia/lymphoma (ATLL) was clinically suspected, and a Southern blot test from peripheral blood was submitted to assess for HTLV-1 monoclonal proliferation.

**Figure 4:**
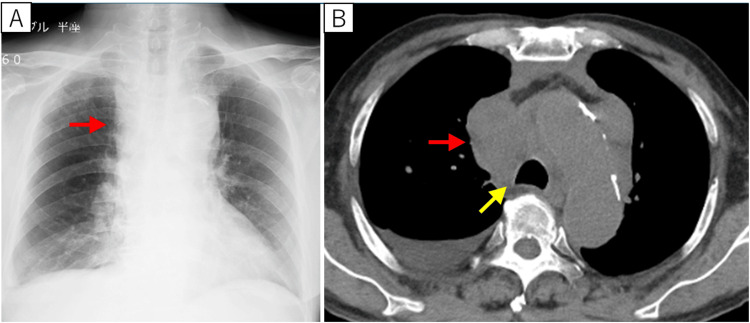
Chest images after palliative radiotherapy to the mediastinum and steroid administration After treatment began, the mediastinal mass lesion was significantly reduced on chest X-ray (A) and chest CT (B) (red arrow), and the compressive narrowing of the trachea improved (yellow arrow) CT: computed tomography

Hematological evaluation, including bone marrow examination and FDG-PET scan, was required to promptly initiate chemotherapy; however, management at nearby hematology departments was difficult. Given the rapid progression of airway stenosis, palliative radiotherapy (1.8 Gy/fraction, total 45 Gy/25 fractions planned) combined with corticosteroids (dexamethasone 4 mg twice daily) was started in our hospital’s Department of Radiology. Following initiation of treatment, the mediastinal lesion showed a marked reduction in size, and steroids were discontinued after six days. Correspondingly, the patient’s dyspnea, facial edema, and bilateral arm swelling improved, and chest imaging revealed a marked decrease in the mediastinal mass (Figures 4A, 4B).

After receiving 12.6 Gy in seven fractions, the patient was transferred to the hematology department. Although the HTLV-1 monoclonality test (Southern blot) using peripheral blood was ultimately negative, a comprehensive assessment of the clinical course, cytological features, and immunohistochemical findings led to a final diagnosis of ATLL. Systemic chemotherapy was initiated at the receiving institution; however, due to the transfer, detailed information regarding the treatment regimen and subsequent clinical course was unavailable.

## Discussion

We reported the case of an elderly patient with a large mediastinal mass caused by malignant lymphoma, who presented with SVC syndrome and rapidly worsening respiratory distress. Palliative radiotherapy combined with steroid administration resulted in significant lesion shrinkage and rapid clinical improvement. The Lugano classification defines “bulky” disease in malignant lymphoma as a lesion measuring approximately 6-10 cm or larger, depending on histological subtype [6]. Common causes of an anterior mediastinal mass include thymoma, teratoma, thyroid disease, and lymphoma, whereas congenital cysts such as foregut cysts and pericardial cysts more commonly occur in the middle or posterior mediastinum. Neurogenic tumors also predominate in the posterior mediastinum [1].

A single-institution retrospective study of 283 anterior mediastinal tumors from 1997 to 2016 in Japan reported the following distribution: 34% thymoma, 16% thymic carcinoma, 3% metastatic tumors, and 27% malignant lymphoma. Among these lymphomas, 13% were primary mediastinal large B-cell lymphoma (PMBL), 9% classical Hodgkin lymphoma (CHL), 2% T-lymphoblastic lymphoma (T-LBL), 1% MALT lymphoma, and 2% were classified as other malignant lymphomas, a category that included ATLL [2]. In the present case, because the lesion was primarily situated in the anterior and middle mediastinum, combined with elevated LDH and IL-2R, malignant lymphoma was suspected from the outset. The cytological findings confirmed lymphoma, and clinical suspicion for ATLL was raised given positive HTLV-1 serology and hypercalcemia.

Treatment strategies for lymphoma manifesting as a large mediastinal mass vary based on the histological subtype. In cases of PMBL with emergent presentations such as SVC syndrome or airway compromise, tissue diagnosis (often including surgical biopsy) followed by systemic chemotherapy is standard. Radiation before tissue diagnosis risks obscuring the histological features and may compromise bone marrow in the sternum, ribs, and spine, thus hindering full-dose chemotherapy [7]. However, these previously reported cases primarily involved younger patients in their 20s to 50s, managed predominantly by hematology departments [8,9,10,11]. To the best of our knowledge, no similar cases involving patients in their 80s have been reported. Our case is significant as it demonstrates appropriate management provided by a respiratory medicine department rather than a hematology department. Furthermore, in ATLL, which is relatively rare, no well-established guideline addresses this specific scenario, and evidence is limited.

In situations where urgent hematology referral is not possible, especially due to the absence of an immediately available hematology department capable of urgent intervention, a respiratory medicine team, although not specialized in hematologic malignancies, has to take prompt action. In such resource-limited settings, the primary goal shifts toward life-saving stabilization rather than strict adherence to standard diagnostic protocols. Early initiation of palliative radiotherapy combined with corticosteroids was chosen as the most feasible and effective interim strategy to reduce the mediastinal mass and relieve airway obstruction. The patient’s advanced age, preserved functional status, and strong desire for active treatment also supported the decision to prioritize immediate symptom control. Although this approach carries risks, including the potential to obscure histopathological findings, it enabled stabilization and safe transfer to a hematology department for definitive diagnosis and systemic chemotherapy.

This case underscores the importance of flexible, multidisciplinary decision-making and the need for interim management strategies when immediate access to specialized hematologic care is unavailable, particularly in urgent, life-threatening situations.

## Conclusions

This report demonstrates that even in very elderly patients with massive mediastinal lymphoma causing SVC syndrome and critical airway compromise, a flexible and prompt management approach can yield significant clinical improvement. When urgent referral to hematology is not feasible, the respiratory medicine department can effectively stabilize the patient through the early initiation of palliative radiotherapy combined with corticosteroid therapy. This strategy resulted in rapid reduction of the mediastinal mass, alleviation of respiratory distress, and safe transfer for definitive systemic chemotherapy. Our experience suggests that an adaptable, multidisciplinary approach is vital in managing complex cases in high-risk patients, and it provides a practical interim solution for similar challenges in resource-limited settings.

## References

[1] Duwe BV, Sterman DH, Musani AI (2005). Tumors of the mediastinum. Chest.

[2] Maeshima AM, Taniguchi H, Suzuki T (2017). Distribution of malignant lymphomas in the anterior mediastinum: a single-institution study of 76 cases in Japan, 1997-2016. Int J Hematol.

[3] Wright K, Digby GC, Gyawali B, Jad R, Menard A, Moraes FY, Wijeratne DT (2023). Malignant superior vena cava syndrome: a scoping review. J Thorac Oncol.

[4] Anai S, Hashisako M, Ikegame S, Wakamatsu K, Nagata N, Nakanishi Y, Kajiki A (2012). Mantle cell lymphoma involvement of the pleura and tuberculous pleurisy with pulmonary tuberculosis: a case report and literature review. J Infect Chemother.

[5] Anai S, Gushiken H, Chinen S, Gakiya A, Kiyuna M (2023). Successful diagnosis by video-assisted thoracoscopic surgical lung biopsy in a case of progressive primary pulmonary extranodal natural killer/T-cell lymphoma, nasal type: a case report. Cureus.

[6] Cheson BD, Fisher RI, Barrington SF, Cavalli F, Schwartz LH, Zucca E, Lister TA (2014). Recommendations for initial evaluation, staging, and response assessment of Hodgkin and non-Hodgkin lymphoma: the Lugano classification. J Clin Oncol.

[7] Arnold S Freedman, Jon C Aster, Jonathan W Friedberg (2025). Primary mediastinal large B-cell lymphoma. https://www.uptodate.com.

[8] Shrestha R, Khadka S, Saunders H, Helgeson S (2025). Diffuse large B-cell lymphoma causing central airway obstruction: a case report. Cureus.

[9] Saraya T, Shimura C, Mikura S (2008). Huge mediastinal mass with SVC syndrome accompanying numerous chest wall collateral vessels. Intern Med.

[10] Rajabto W, Priantono D (2020). Primary CD20-positive mediastinal diffuse large B-cell lymphoma. Respirol Case Rep.

[11] Besteiro B, Teixeira C, Gullo I, Pereira S, Almeida M, Almeida J (2021). Superior vena cava syndrome caused by mediastinal lymphoma: a rare clinical case. Radiol Case Rep.

